# A case report of metastatic giant sarcomatoid melanoma with BRAF V600E mutation: a complete response to targeted therapy

**DOI:** 10.18632/oncotarget.27701

**Published:** 2020-08-25

**Authors:** Matteo Torresetti, Donatella Brancorsini, Francesca Morgese, Valeria Cognigni, Alessandro Scalise, Rossana Berardi, Giovanni Di Benedetto

**Affiliations:** ^1^Clinic of Plastic and Reconstructive Surgery, Marche Polytechnic University Medical School, Ancona, Italy; ^2^Section of Pathological Anatomy, Marche Polytechnic University Medical School, Ancona, Italy; ^3^Clinic of Medical Oncology, Marche Polytechnic University Medical School, Ancona, Italy

**Keywords:** sarcomatoid melanoma, giant melanoma, BRAF mutation, targeted therapy, next-generation sequencing

## Abstract

Sarcomatoid melanoma is an extremely rare pattern of malignant melanoma, and only few cases have been described throughout the literature. We herein report a case of a patient with newly diagnosed, metastatic giant sarcomatoid melanoma of the arm. The patient underwent surgical removal of the huge mass, and NGS sequencing demonstrated BRAF V600E mutation.

In view of histological, immunohistochemical and molecular findings, a combined BRAF/MEK inhibitor (BRAF/MEK-i) therapy was prescribed as first line treatment. A complete response (over one year) to targeted therapy was obtained, and no adverse events have been reported. The patient maintained a full range of shoulder and elbow movements, and she is able to live independently and resume her daily activities. We therefore recommend that all patients with undifferentiated melanomas, sarcomatoid cutaneous malignancies or other mesenchymal tumours, should undergo BRAFV600E mutation testing.

## INTRODUCTION

Giant cutaneous melanomas have rarely been described in the literature; the term “giant”, although not formally defined, has been previously proposed for those lesions having a diameter larger than 10 cm [1].

Malignant melanomas show a wide variety of cytomorphological features, architectural patterns and stromal changes; hence may mimic several non-melanocytic tumours such as carcinomas, lymphomas, sarcomas, benign stromal tumours, plasmacytomas and germ cell tumours [2]. Malignant melanoma exhibiting a prominent sarcomatoid component, or sarcomatoid melanoma, is a further exceedingly rare pattern of melanoma with very few cases previously reported. Moreover, immunohistochemical evidence of melanocytic differentiation may be lacking.

Hence, it may pose several diagnostic conundrums as it encompasses a broad differential diagnosis of primary cutaneous pleomorphic spindle-cell neoplasms [3].

The exceptional rarity of these tumours and the existing scarce literature regarding its clinicopathologic behaviour, create significant diagnostic and therapeutic challenges. Therefore, the efficacy of targeted immunotherapies recently approved for melanoma is unknown.

We report our experience in treating a patient that had BRAFV600E mutant giant sarcomatoid melanoma of the arm, that was successfully treated with a combined BRAF/MEK inhibitor (BRAF/MEK-i) therapy after surgical removal of the tumour. We performed a literature search and did not identify any reported case of BRAF V600E mutant sarcomatoid melanoma patients treated with BRAF-i target therapy.

The authors believe that the present report represents a peculiar and extraordinary rare case both for its clinical presentation (including its exceptional size), diagnostic challenges and clinical behaviour.

## CASE PRESENTATION

**Figure 1:**
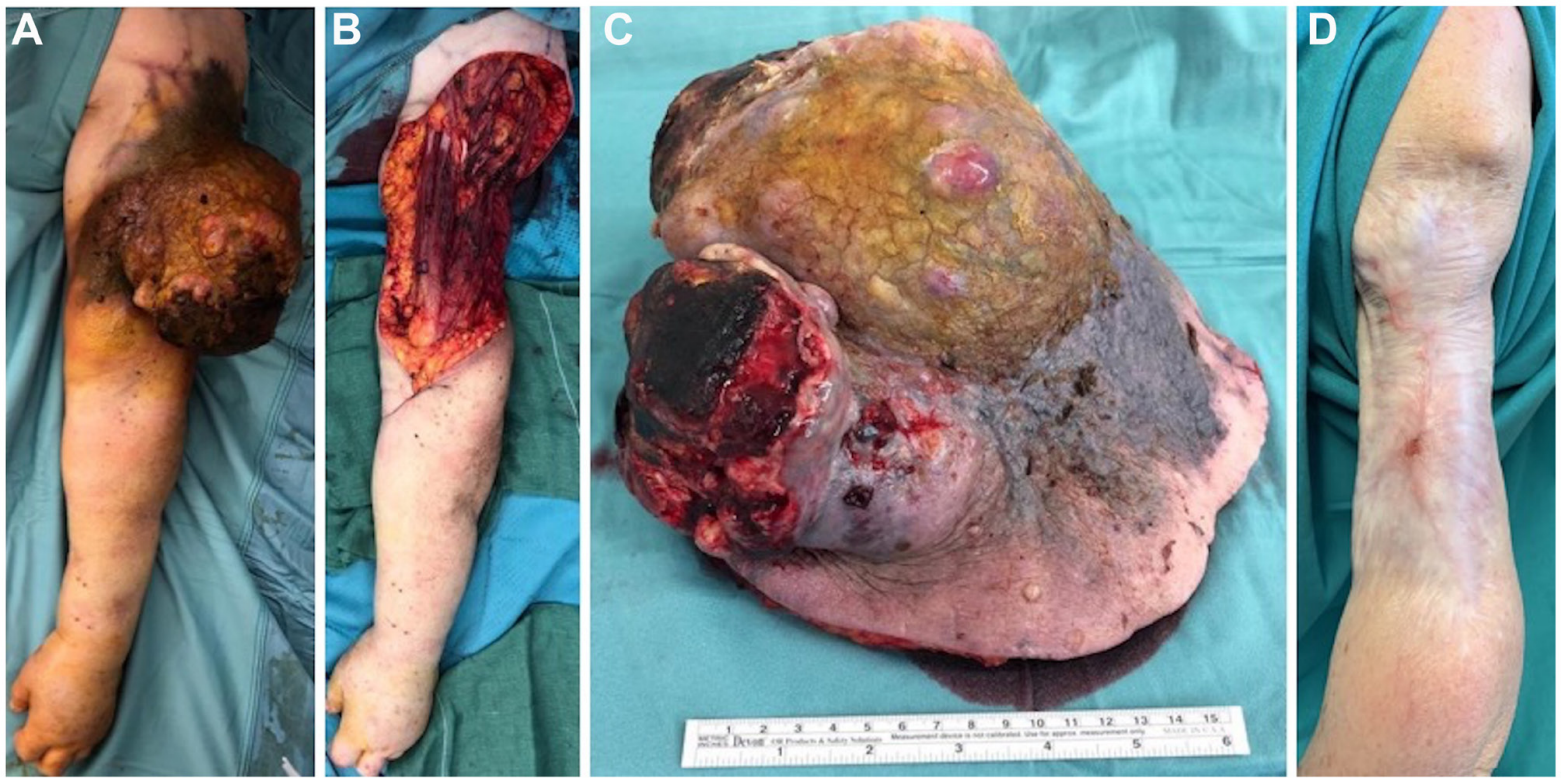
Initial presentation, surgical procedure and postoperative outcome. Preoperative picture of the huge tumor (**A**); intraoperative figure showing the defect after tumor excision (**B**) and the sample sent to pathologist, measuring 19 × 16 cm (**C**); one-year follow-up picture showing no recurrences of the disease and an acceptable aesthetic result (**D**).

A 70-year-old female was referred to the emergency department of our hospital for a large bleeding mass on the lateral aspect of her left arm measuring 19 × 16 cm (Figure 1). The lesion had been present for the last 10 months but the patient had no sought for medical attention, and it was finally reported by the woman for increasing fatigue and continuous bleeding of the lesion. Physical examination revealed a multilobed exophytic and vegetating mass, with several ulcerated, necrotic and pigmented areas. She had no sensory, motor or vascular deficits, and had a full range of shoulder and elbow movements. No axillary palpable lymphadenopathies were clinically detected. Blood test findings revealed severe iron deficiency anemia (Hb 7.0 gr/dl) and hypoproitenemia (Albumin 1.5 gr/dl) due to intermittent bleeding of the tumour and serum loss. Preoperative incisional biopsies of the lesion suspected a diagnosis of malignant melanoma, as immunohistochemical stains demonstrated the tumour cells to be negative for MART-1, HMB-45, LCDA and CD138, while they were positive for S100 protein and Vimentin.

An initial staging whole-body computed tomography (CT) scan showed several enlarged lymph nodes in the left groin, but no other evidence of distant metastases. These were biopsied and found to be benign. Therefore the patient underwent wide local excision of the mass with a 2 cm margin which included fascia and a cuff of the deltoid muscle bellies after a multidisciplinary tumour board consultation, and an immediate reconstruction by using a dermal substitute (Integra^®^ Dermal Regeneration Template) was performed. Postoperative period was uneventful and the patient was discharged after 6 days. The patient was then followed in outpatient for wound management.

### Histological examination

Grossly, the neoplasm was a huge pedunculated and extensively ulcerated mass.

Histological examination revealed a highly cellular neoplasm occupying dermis and hypodermis with a diffuse pattern of growth, showing a variable admixture of atypical epithelioid and spindle cells, with a predominance of the epithelioid component.

**Figure 2:**
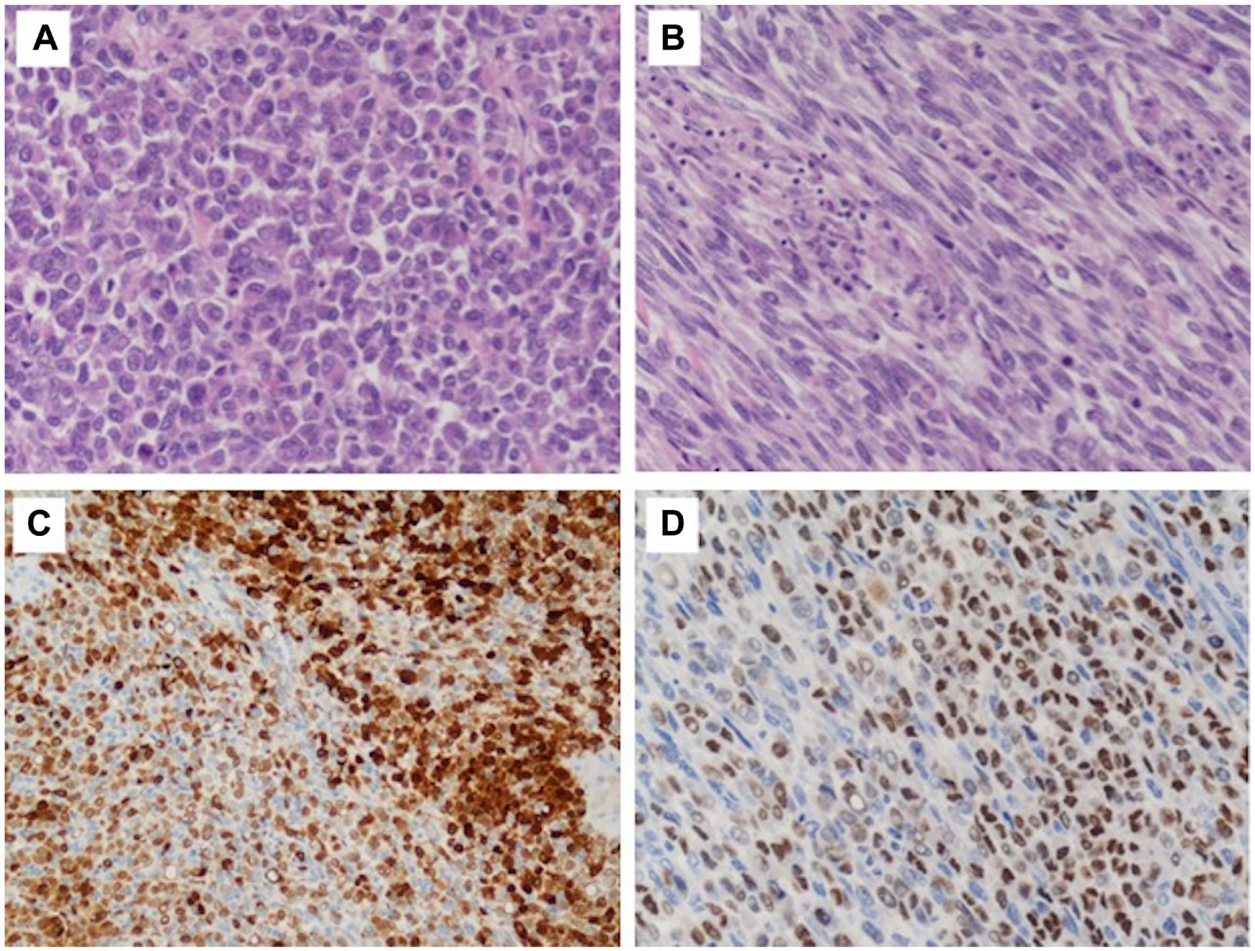
Histological findings of the tumor. High power view (H&E, 20× magnification); tumor cells were relatively uniform with eosinophilic cytoplasm, nuclear pseudoinclusions and prominent nucleoli (**A**–**B**). Immunohistochemical staining demonstrated strong but focal positivity for S-100 protein (**C**) and SOX10 (**D**).

Tumour cells were relatively uniform with eosinophilic cytoplasm, nuclear pseudo-inclusions and prominent nucleoli (Figure 2A and 2B). Mitotic figures were readily found and tumour necrosis were frequently observed. There was no evidence of a junctional melanocytic component.

Immunohistochemical stains demonstrated strong but focal positivity for S-100 protein and SOX10 (Figure 2C and 2D) and negativity for melanocytic (Melan-A, HMB45), vascular (CD31, ERG) and myogenic markers (smooth muscle actin, desmin).

Subsequent testing by Ion Torrent next-generation sequencing (NGS) using the Cancer Hotspot Panel v2 (ThermoFisher Scientific, Life Technologies) revealed the presence of p. V600E BRAF mutation involving the exon 15, and a p. R80STOP mutation of CDKN2A in 53% and 85% of the analysed DNA, respectfully. The mutational analysis was performed by an outside institution.

Therefore, histological, immunohistochemical and molecular findings suggested the diagnosis of giant sarcomatoid melanoma.

Wound healing by secondary intention within 4 months was obtained. One year later, punch biopsies were performed for a hard-to-heal small wound on the left arm, but no signs of recurrent disease were detected.

### Combined target therapy

**Figure 3:**
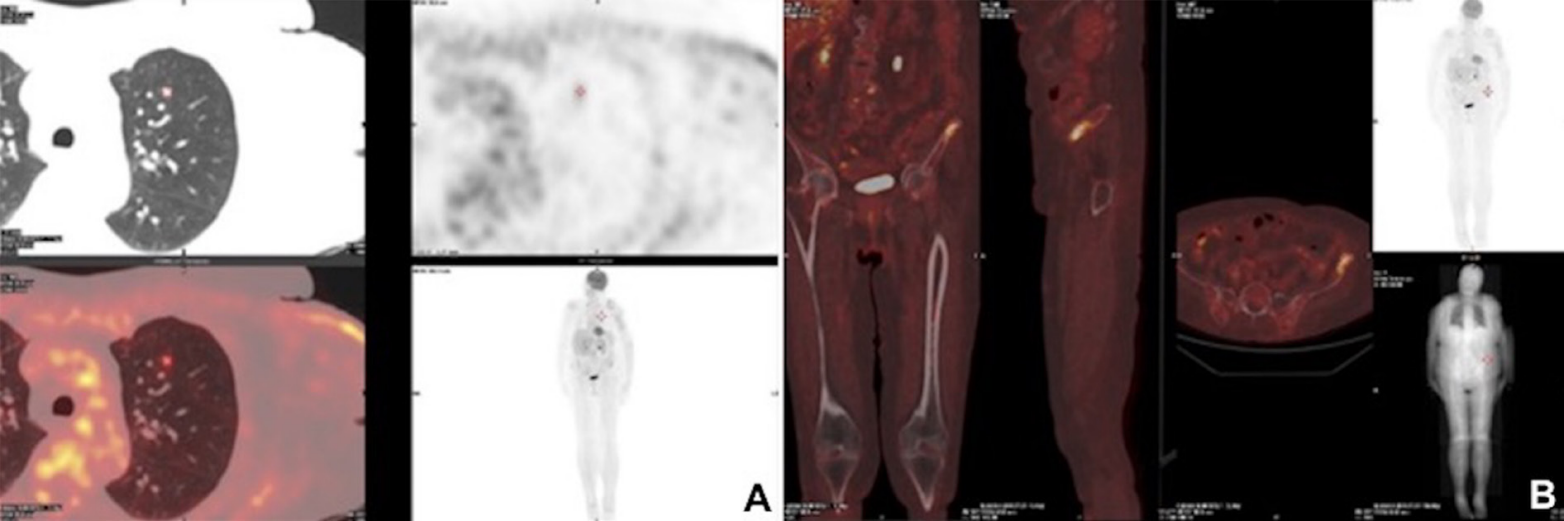
Postoperative radiological images. Three-months postoperative PET/CT scan showing a FDG-avid, presumptive pathological uptake in left lung (SUV max = 2.0) (**A**) and in left iliac wing (SUV max = 7) (**B**).

Three months post-operatively, whole-body PET/CT scan revealed a FDG-avid, presumptive pathological uptake in left lung (SUV max = 2.0) and in left iliac wing (SUV max = 7) (Figure 3). Considering the differential diagnosis, the stage and the presence of BRAF mutation, the patient appeared candidate to target therapy with BRAF-i in combination with MEK-i. This approach was shared by a multidisciplinary tumour board consultation. After fulfillment of normal echocardiography and optical coherence tomography scan, the patient started Dabrafenib 150 mg bid and Trametinib 2 mg od 4 months post-operatively. Target therapy was well tolerated. Imaging with whole-body CT scan after 4 months of combined target therapy revealed partial response in both sites of disease. In particular, there was a significant reduction in the lung metastasis (0.3 cm vs. 1 cm) and osteosclerosis of the left wing.

**Figure 4:**
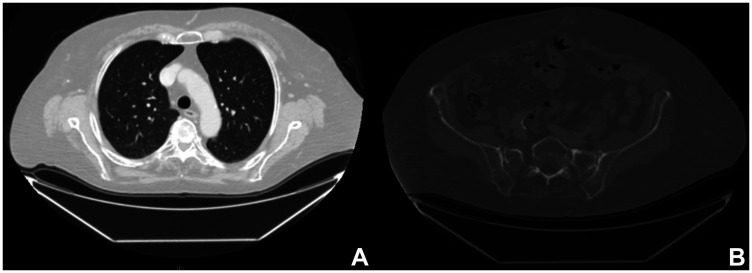
Radiological images after combined targeted therapy. Computed tomography one year after BRAF/MEK-i therapy, showing a complete response of the disease with regression of the lung (**A**) and bone metastasis (**B**).

**Figure 5:**
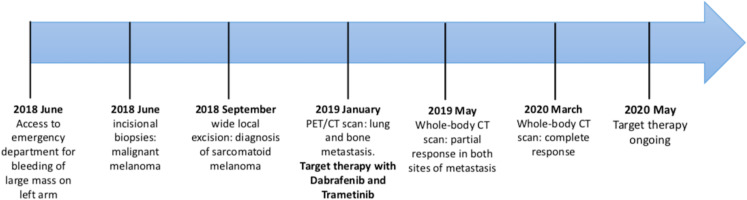
Timeline of patient’s medical history.

One year later, the whole-body CT scanning demonstrated a complete response of the disease with regression of the lung and bone metastasis (Figure 4). Currently, the treatment with Dabrafenib and Trametinib is ongoing at full dose with an optimal tolerance (completed XV cycle). Clinically, the patient maintained a full range of shoulder and elbow movements, and she is able to live independently and resume her daily activities. Figure 5 summarizes the whole medical history of the patient.

## DISCUSSION

Sarcomatoid melanoma is an extremely rare pattern of malignant melanoma, and only few case reports or case series have been described. According to Erstine et al. [3], until 2017 had been reported only 3 cases of sarcomatoid melanoma. The first two reported cases exhibited a sarcomatoid phenotype in lymph node metastatic deposits, but not in the primary cutaneous tumour [4, 5], while Kiuru et al. [6] and Erstine et al. [3] reported the first cases of primary cutaneous sarcomatoid melanoma described in the literature. Afterwards, two more cases of primary cutaneous sarcomatoid melanoma [7, 8] have been reported (Table 1).

**Table 1:** Reported cases in the literature of sarcomatoid melanoma

Author/Year	No of cases/Site of primary tumour	Age (years)/Sex	Size (cm) of primary tumour/ Metastatic deposit	Sarcomatoid phenotype/Molecular profile	Nodal/Extranodal diffusion	Therapy	Outcome
Banerjee S, et al./1996 [4]	1/Epigastric area	54/F	NA/4.5 × 2.5 × 2.0	Lymph node metastatic deposits (axilla)/NA	Axillary, groin, iliac lymph nodes/subcutaneous metastatis	Removal of metastasis + groin dissection + decarbazine + interferon	Alive (9 years follow-up)
Kacerovska D, et al./2009 [5]	1/Heel	63/M	NA/7 × 6.5 × 3.5	Lymph node metastatic deposits (groin)/NA	Groin metastasis	Removal of metastasis	Died (multiple liver and bone metastases)
Kiuru M, et al./2014 [6]	1/Scalp	66/M	NA	Primary cutaneous SM/NA	NA	NA	NA
Erstine EM, et al./2017 [3]	2/Breast Heel	65/F	1,8/1	Primary cutaneous SM/BRAF mutational analysis: negative	Axillary metastasis	Wide local excision + SLNB + axillary dissection + interferon	PFS: 9 months Died after 19 months
		62/M	NA	Primary cutaneous SM/BRAF mutational analysis: negative	Groin metastasis	Wide local excision + SLNB + superficial inguinal dissection	PFS: 25 months
Fraga GR/2017 [7]	1/Scalp	75/M	1,2 × 1/NA	Primary cutaneous SM	NA	NA	NA
Lefferts JA, et al./2020 [8]	1/Thigh	73/M	6 × 5 × 3/NA	Primary cutaneous SM/NRAS-BRAF mutational analysis: positive (*NRAS* p. Q61L)	Inguinal metastasis	Pembrolizumab	PFS: 3 months

Abbreviations: NA: not applicable; SM: sarcomatoid melanoma; SLNB: sentinel lymph node biopsy; PFS: progression free survival.

To the best of our knowledge, this case represents the first giant primary cutaneous sarcomatoid melanoma described in the literature, and it is also one of the largest upper limb melanomas reported worldwide. By an accurate analysis of the reported cases, we can assert that no guidelines exist both for diagnostic and therapeutic management of this extremely rare tumour.

Hotspots mutations of the oncogenes BRAF and NRAS are the most common genetic alterations in cutaneous melanoma, and BRAF-targeted therapies demonstrated significant clinical benefits. Furthermore, several reports have anecdotally documented BRAF mutation in 1–9% of all sarcomas as highlighted by Cipriani et al. [9], and the same authors suggested that BRAF mutational analysis should be considered in those patients with a spindle cell malignancy and a history of melanoma, as a positive result may indicate de-differentiated melanoma.

From a therapeutic perspective, when a target and its specific inhibitor exist, the target therapy shows high response rate, usually, independently of histotype [10–15] (Table 2).

**Table 2:** Reported cases in the literature of BRAF mutant sarcoma patients treated with BRAFi target therapy

Author/year	Age (years)/sex	Diagnosis	Site of primary tumour	Site of metastasis	Target therapy	Response	Outcome
Kaplan HG/2013 [10]	51 F	Malignant peripheral nerve sarcoma	Right axilla	Thoracic/abdominal subcutaneous + local and adrenal recurrence	Vemurafenib as second line of treatment	Partial response after 1 month	Unknown
Idbaih A, et al./2014 [11]	40 M	Histiocytic sarcoma	CNS		Vemurafenib as first line of treatment	Partial response	PFS: 4 months
Protsenko SA, et al./2015 [12]	46 M	Clear cell sarcoma	Lumbar area	Local recurrence, bone and lung	Vemurafenib as second line of treatment	Complete response after 8 weeks	Unknown
Mitsis D, et al./2015 [12]	69 M	high-grade spindle cell STS	Abdomen-pelvis	Lung	Dabrafenib and trametinib as second line of treatment	Partial response after 6 weeks	Unknown
Branco B, et al./2019 [14]	22 F	Histiocytic sarcoma	NA	abdominal lymph-nodes and liver	Vemurafenib (+Gemcitabine) as second line of treatment	Complete response after 18 months	Treatment ongoing at time of submission
Watanabe S, et al./2020 [15]	23 F	Synovial sarcoma	Superior mediastinum	Local recurrence	Dabrafenib + Trametinib as second line of treatment	Complete response at 3 months after starting	PFS: 7,5 months (NRAS mutation as mechanism of resistance)

Abbreviations: NA: not applicable; CNS: central nervous system; STS: soft tissue sarcoma; PFS: progression free survival.

Due to these findings, the proposal to perform BRAF mutational analysis in rare tumours such as sarcoma appeared a reasonable strategy for initiating appropriate clinical management. In previous reports, these mutations were rarely encountered in sarcomatoid melanoma, and only Lefferts et al. recently reported a somatic variant of NRAS p. Q61L revealed by NRAS-BRAF Mutation Assay in the sarcomatoid component [8]. Erstine et al. also performed BRAF mutational analysis of the tumour but no mutations were detected [3].

The present case reported the first exceptional therapeutic response to first-line combined BRAF and MEK-targeted therapy in a giant metastatic sarcomatoid melanoma harboring BRAF V600E mutation. This tumour was exceptionally responsive to targeted treatment, with a complete radiological and clinical response after a few months.

## CONCLUSIONS

Our findings suggest that first line combined treatment with BRAF and MEK inhibitors can lead to dramatic tumour response in patients with *BRAF*V600E metastatic sarcomatoid melanoma. We therefore recommend that all patients with undifferentiated melanomas, sarcomatoid cutaneous malignancies or other mesenchymal tumours, should undergo BRAFV600E mutation testing, in order to guide the clinicians in the differential diagnosis, thus ensuring the most appropriate treatment for the patient.
